# Health education through mass media announcements by loudspeakers about malaria care: prevention and practice among people living in a malaria endemic area of northern Myanmar

**DOI:** 10.1186/s12936-019-2985-6

**Published:** 2019-11-12

**Authors:** Pyae Linn Aung, Tepanata Pumpaibool, Than Naing Soe, Jessica Burgess, Lynette J. Menezes, Myat Phone Kyaw, Liwang Cui

**Affiliations:** 1 College of Public Health Sciences, Chulalongkorn University, Bangkok, 10330 Thailand; 2 Department of Public Health, Ministry of Health and Sports, Naypyitaw, Myanmar; 3 Division of Infectious Diseases and International Medicine, Department of Internal Medicine, Morsani College of Medicine, University of South Florida, 3720 Spectrum Boulevard, Suite 304, Tampa, FL 33612 USA; 4 Myanmar Health Network Organization, Yangon, Myanmar

**Keywords:** Announcement, Loudspeaker, Malaria infection, Malaria care, Rural Myanmar

## Abstract

**Background:**

Interventions to raise community awareness about malaria prevention and treatment have used various approaches with little evidence on their efficacy. This study aimed to determine the effectiveness of loudspeaker announcements regarding malaria care and prevention practices among people living in the malaria endemic villages of Banmauk Township, Sagaing Region, Myanmar.

**Methods:**

Four villages among the most malaria-burdened areas were randomly selected: two villages were assigned as the intervention group, and two as the control. Prior to the peak transmission season of malaria in June 2018, a baseline questionnaire was administered to 270 participants from randomly selected households in the control and intervention villages. The loudspeaker announcements broadcasted health messages on malaria care and prevention practices regularly at 7:00 pm every other day. The same questionnaire was administered at 6-month post intervention to both groups. Descriptive statistics, Chi-square, and the t-test were utilized to assess differences between and within groups.

**Results:**

Participants across the control and intervention groups showed similar socio-economic characteristics; the baseline knowledge, attitude and practice mean scores were not significantly different between the groups. Six months after the intervention, improvements in scores were observed at *p*-value < 0.001 in both groups, however; the increase was greater among the intervention group. The declining trend of malaria was also noticed during the study period. In addition, more than 75% of people expressed positive opinions of the intervention.

**Conclusions:**

The loudspeaker intervention was found to be feasible and effective, as shown by the significant improvement in scores related to prevention and care-seeking practices for malaria as well as reduced malaria morbidity. Expanding the intervention to a larger population in this endemic region and evaluating its long-term effectiveness are essential in addition to replicating this in other low-resource malaria endemic regions.

## Background

Globally, 219 million people contracted malaria in 2017, with an estimated 435,000 deaths [1]. Although sub-Saharan Africa accounted for 92% of cases and 93% of all malaria deaths, the Southeast Asian region represented 70% of the remaining cases and deaths [1]. In 2015, the World Health Organization (WHO) developed the strategy to eliminate malaria in the Greater Mekong Subregion (GMS) by 2030 [2]. By 2016, Myanmar had the highest annual parasite incidence rate among the GMS countries [1]; 291 out of 330 townships in Myanmar were endemic with more than 40 million people at risk for malaria. However, the number of deaths and cases of malaria in Myanmar had dropped significantly from 1707 and 516,041 in 2010 to 21 and 105,178 in 2016, respectively [3]. Yet, high-level malaria transmission persisted in hard-to-reach rural areas. With strong government commitment and under the leadership of the national malaria control programme, a roadmap to eliminate malaria by 2030 was announced in Myanmar [3, 4].

Effective prevention and prompt care-seeking practices are essential to halt the transmission of malaria. One of the core preventive measures is the use of long-lasting insecticide-treated nets (LLINs). However, low levels of LLIN utilization have been noted among communities from malarious regions of Myanmar [5]. Thus, population behaviour toward proper LLIN usage should be addressed together with adequate LLIN distribution. In addition, early diagnosis and timely treatment are crucial [6], as treatment delays may lead to severe manifestations of the disease within a few hours or days [7, 8]. Ideally, malaria treatment should commence within 24 h after the onset of fever, and self-treatment is not recommended [9–11]. Delays in treatment-seeking have been documented in malaria patients from various endemic areas. One study in Thailand found that 79.4% of patients delayed in seeking malaria treatment [12]. Some studies identified economic (family income and financial difficulties), geographical (distances from a health facility), and health-system factors, as determinants of the delay in accessing treatment [13, 14]. Therefore, improvement in care-seeking behaviour is needed for effective management of malaria cases.

As of late 2017, 24 different organizations conducted 30 malaria-related health education intervention projects in scattered malaria hotspot areas across Myanmar [15]. Consistently low levels of community knowledge, attitude, and practice (KAP) were observed in many studies conducted in Myanmar [16–18]. According to relevant studies, communities were aware of the recommended health facts, but the majority continued to follow their own intervention methods [19, 20]. To overcome this lack of follow-through, designing simple health messages that are delivered repeatedly are needed. Evaluation results from many of these health-related interventions are lacking and, therefore, studies of innovative activities that incorporate an evaluation component are essential. For the effectiveness of the education programmes, not only the intervention itself, but the demographic characteristics such as age, gender, family size and income, and education level should be considered [21, 22]. In addition, traditional health-talks and community-based interventions delivered through voluntarily recruited villagers should be considered for whole population coverage in malaria prevention campaigns.

Loudspeakers are widely used in Myanmar for various purposes and occasions to advise the community, especially in rural areas. Local officials can use these easily, irrespective of government electricity services or space availability. Simple setup included an amplifier, microphone, and a pair of horns attached to two units. For the power source, 160–220 volts are required, supplied either by the government line or another power source such as inverters, solar system, and generators. One study reported that loudspeaker announcements are effective and conducive to the spread of information in real time [23]. Moreover, it could inform the community about a disease outbreak and ensure emergency preparedness immediately with minimum efforts and without a delay. In this study, the effectiveness of health messages announced through loudspeakers regarding malaria care-seeking and prevention practices was evaluated among people living in malaria endemic areas of Myanmar.

## Methods

### Village settings

**Fig. 1:**
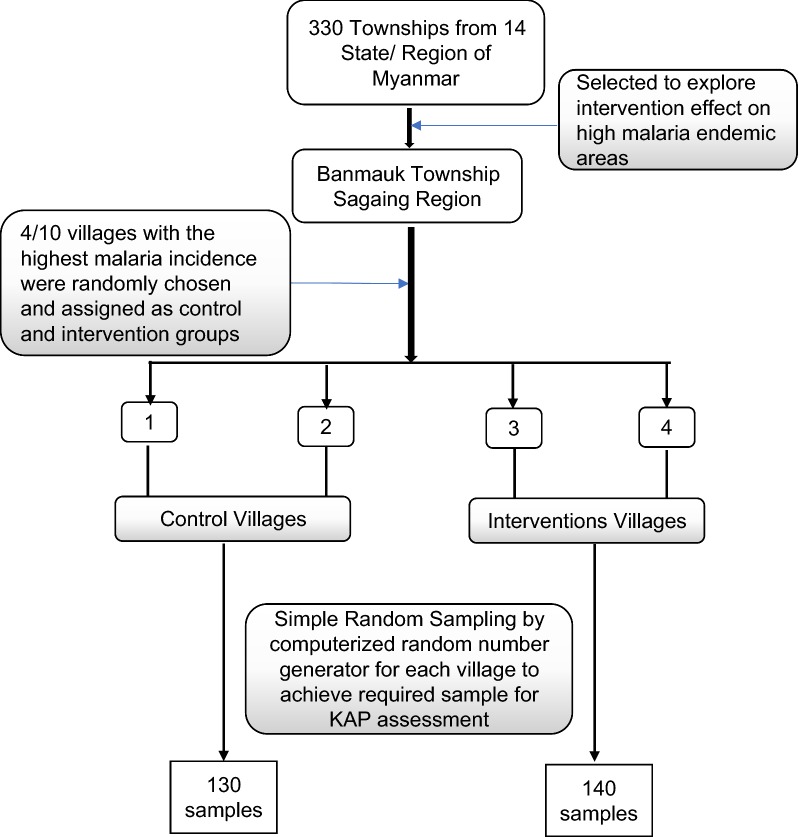
Scheme of selection of malaria endemic villages and villager populations

This study was implemented in Banmauk, a malarious township located in Sagaing Region of northern Myanmar. The Banmauk Township was purposely selected because of its high malaria prevalence. In 2017, Banmauk had an annual parasite incidence (API) of 26.62/1000 as compared to the overall country API of 1.64/1000. From the top 10 villages based on their ranking in the 2017 malaria incidence, two were selected for the intervention, and another two served as the control to follow a quasi-experimental study design (Fig. 1). The intervention and control villages consisted of a total of 300 and 310 households, with a total population of 2460 and 2170, respectively. These four villages are located more than 20 km from the downtown area of Banmauk. There was a buffer zone of at least 5 km between each pair of the selected villages. A trained village malaria worker (VMW) supported by the township vector-borne disease control (VBDC) team was assigned to each village. All four VMWs had elementary school education with 4 years of experience as a VMW. The VBDC team provided 50,000 MMK (~ 35 USD) quarterly to the VMWs as a travel incentive. VMWs received two to three refresher trainings annually, which were facilitated by the township VBDC team. The training content included general knowledge of malaria, diagnosis and treatment of malaria, timely referral, and practice sessions. Supplies for malaria diagnosis and treatment were provided to the VMWs. VMWs passively monitored the malaria cases in their assigned villages. For each patient examined, a standard case report form was filled, which was subsequently gathered and compiled by the township VBDC team.

The study protocol was reviewed and approved by the Institutional Technical and Ethical Review Board, University of Public Health, Yangon, Myanmar and the Pennsylvania State University Institutional Review Board.

### Health message announcement activity

**Fig. 2:**
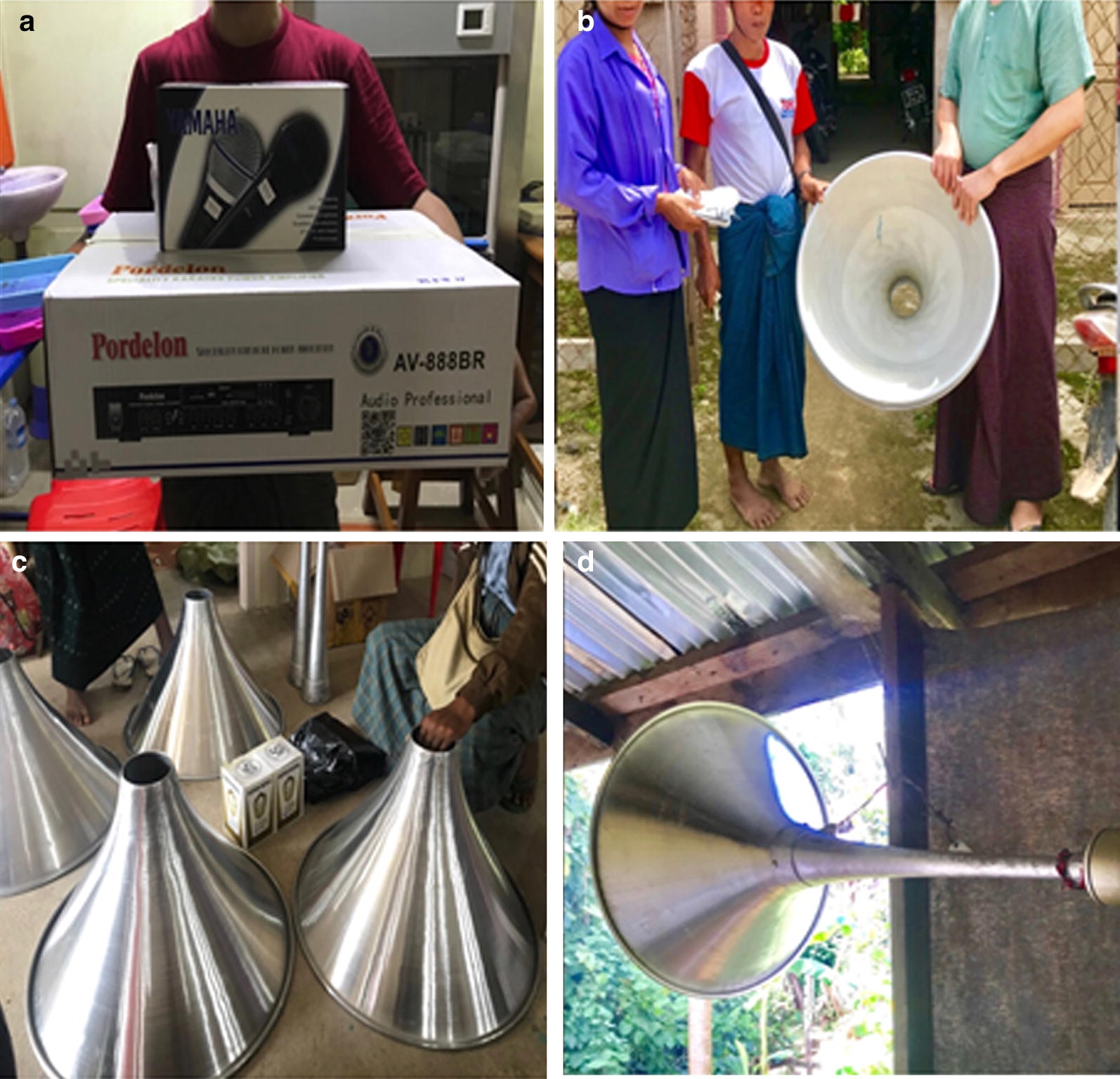
Setting-up of the loud-speaker system. **a** Amplifiers and microphones for the loud-speakers. **b** Transfer of the materials to the villages. **c** Materials for the system setting-up. **d** A loud-speaker system set ready for announcement

The study team conducted an informal advocacy meeting with local authorities, together with the respective VMW in each intervention village, and briefly explained the health message announcement activity. A full set of loudspeakers was provided directly to each intervention area. The loudspeakers were installed at each volunteer’s home, and the volume was guaranteed to cover the whole village (Fig. 2). The researcher interviewed three households in the immediate surrounding as well as the peripherals of the villages about audibility and potential annoyance, to decide the places to put the loudspeakers and to adjust the volumes. Next, VMWs received 2 h of on-site training and were given 10 sentences of standardized health messages. These sentences were adapted from the malaria section of a published booklet of the Ministry of Health and Sports (MoHS) called ‘*Standardized Health Messages 2018*’ [24], targeting malaria-like symptoms, diagnosis and treatment, mainly for care-seeking behaviours and treatment adherence. The ‘Loudspeaker-based Announcement’ intervention programme was implemented in June 2018, whereby announcements were repeated in the evening (at approximately 7:00 pm) every other day until November 2018. During this six-month period, malaria-positive cases were identified by the assigned VMWs through passive case detection. To ensure smooth and timely operation of the activities, the respective village authorities were routinely contacted by the research team.

### Questionnaire

To explore KAP levels within this community, a standard questionnaire was prepared in Myanmar language adapted from the WHO malaria indicator survey [25], which was previously used in Myanmar for a nationwide survey in 2015. Specifically, relevant items were regrouped under each KAP section. The knowledge section was presented in two separate components, pertaining to signs and symptoms of malaria, and diagnosis and treatment of malaria. Most of the questions required participants to select single or multiple responses from a predefined list of options, whereby a score of ‘1’ was assigned for a correct response and ‘0’ for an incorrect response. There were 10 questions in each component and the maximum scores for the two components are ‘20’. The attitude section also comprised two components: severity of malaria and transmission of malaria, for which eight structured mini-statements required a response on a 4-point Likert scale. The practice section was similar to the knowledge section, comprised of ten predefined choice questions under each category regarding proper utilization of LLINs, malaria care-seeking, and treatment adherence. Again, ‘1’ was assigned for a correct response and ‘0’ for a wrong response. Participants in the intervention groups were required to complete an additional questionnaire section to assess their optimism towards delivered announcement activities during the study.

### Sample size calculation

The required sample size for the quasi-experimental study design was calculated by the following means difference formula [26].

$$n = \frac{{2(Z_{\alpha /2} + Z_{\beta} )^{2} \times \sigma^{2} }}{{\Delta^{2} }}$$

where n = sample size, Z_α/2_ = percent under the normal curve at 0.05 = 1.96, Z_β_ = 0.84, σ = standard deviation of malaria prevention practice score after community-directed educational intervention (determined to be 1.98 in a previous study) [27], and Δ = means difference of malaria prevention practice scores after community-directed educational intervention (predetermined to be 0.76 in the previous study) [27]. Accordingly, the sample size was determined to be 105 for each group. After adding 10% of refusal to participate and 20% for dropout, the minimum number of the study population was 135 in each group, representing about 45% of total household leaders in the four study villages.

### Participant selection

Household leaders/members older than 18 years of age who were well informed about their socioeconomic status and overall health were selected to participate in the study. A systematic, random sampling design using a random number generator was used to select the households from the four villages, using a local household list. The houses in each village were identified by the global positioning system (GPS) coordinates, and the household list was divided into four geographical sections. To avoid clustering of selected houses, each section was sampled proportionally. The sampling occurred separately for each pre- and post-intervention survey in the four villages. Hence, although the numbers of participants were the same, the subjects were partially different each time. After signing informed consent, participants were asked to complete an interviewer-administered face-to-face questionnaire before the start of the intervention in May 2018 and upon its completion in November 2018, to compare the scores and identify necessary changes. Three research assistants were hired and trained in human subject research ethics and to deliver the questionnaire.

### Data analysis

Malaria incidence was obtained from the township-level VBDC database, and was utilized to ascertain differences among villages. The dataset included patients’ demographic information as well as the blood test results by a rapid diagnostic test. The malaria diagnostic test result was considered as one of the outcomes of this study. Since individuals who present with fever have the right to seek malaria diagnosis and treatment from VMWs, the nearest health centre, or at the township hospital, free of charge, the township-level data of each village were compiled to avoid missing cases. All data were entered into a Microsoft Excel spreadsheet and then encoded and analyzed using Statistical Package for the Social Sciences (SPSS version 23). Descriptive statistics, followed by Chi-squared and unpaired samples t-tests for statistical differences, were employed with a *p*-value of less than 0.05 considered statistically significant. Within-group comparisons, as well as between group comparisons, were conducted for both pre- and post-intervention periods.

## Results

### Demographics of the study population

Participants’ sociodemographic characteristics were similar between the control and intervention groups. More than half the respondents were 18 to 35 years old, with a similar number of males and females (Table 1). The majority of participants in both groups reported malaria episodes before participation in this study. Few households reported insufficient ownership of LLINs for the family. Family income was reported as adequate by most participants in both groups with more than half of them reporting primary-level education.

**Table 1:** Comparison of socio-demographic characteristics between intervention and control groups

Characteristics	Before intervention		After intervention		*p*-value			
					Within groups		Between groups	
	Control (n = 130)	Intervention (n = 140)	Control (n = 130)	Intervention (n = 140)	Control	Intervention	Before	After
	n (%)	n (%)	n (%)	n (%)				
Age (years)								
18–35	79 (60.7)	77 (55.0)	81 (62.3)	81 (57.9)	0.799	0.181	0.388	0.456
> 35	51 (39.3)	63 (45.0)	49 (37.7)	59 (42.1)				
Sex								
Male	61 (46.9)	75 (53.6)	70 (53.8)	73 (52.1)	0.264	0.811	0.275	0.779
Female	69 (53.1)	65 (46.4)	60 (46.2)	67 (47.9)				
Malaria episodes before study								
Yes	74 (56.9)	88 (62.9)	65 (50.0)	82 (58.6)	0.263	0.463	0.320	0.158
No	56 (43.1)	52 (37.1)	65 (50.0)	58 (41.4)				
Sufficient ownership of LLINs for family members (2 persons/net)								
Yes	126 (96.9)	135 (96.4)	124 (95.4)	131 (93.6)	0.519	0.200	0.821	0.516
No	4 (3.10)	5 (3.6)	6 (4.60)	9 (6.4)				
Enough annual family income								
Yes	109 (83.8)	121 (86.4)	116 (89.2)	124 (88.6)	0.203	0.588	0.551	0.863
No	21 (16.2)	19 (13.6)	14 (10.8)	16 (11.4)				
Education level								
≤ Primary	71 (54.6)	90 (64.3)	72 (55.4)	84 (60.0)	0.901	0.460	0.106	0.443
> Primary	59 (45.4)	50 (35.7)	58 (44.6)	56 (40.0)				

Degree of freedom for Chi-square test = 1, LLIN = Long-lasting insecticide treated net, education: primary—able to read and write or grade 1 to 5

### Comparison between control and intervention groups

**Fig. 3:**
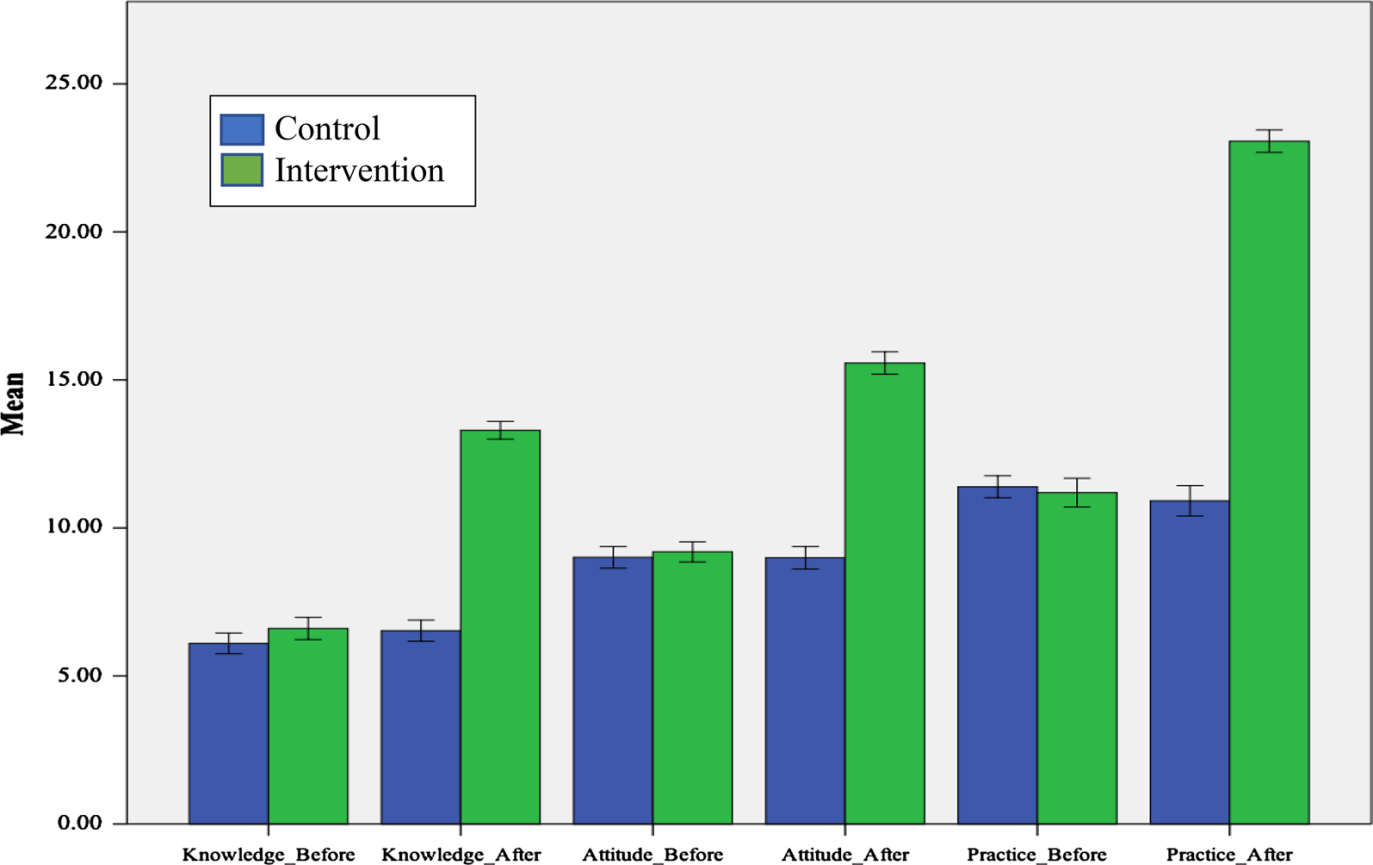
Comparison of KAP scores in the control and intervention villages

To ascertain changes in the overall KAP scores following the six-month intervention, the mean scores and corresponding standard deviations (SD) of individual KAP components for the intervention group were compared with those of the control group (Fig. 3). A large and statistically significant increase in the overall mean score was noticeable in the intervention communities.

The mean scores of each KAP component were compared (Table 2). For the knowledge section, the results revealed no significant difference among the groups prior to the intervention, but a statistically significant difference between the groups post-intervention in knowledge for signs and symptoms of malaria (mean ± SD 3.46 ± 1.30 in the control group versus 6.81 ± 1.43 in the intervention group, *p* < 0.001), and for the section on diagnosis and treatment of malaria (3.07 ± 1.44 in the control group versus 6.49 ± 1.28 in the intervention group, *p *< 0.001).

**Table 2:** Mean scores of knowledge, attitude and practice between two groups before and after intervention

Descriptions	Before intervention				After intervention			
	Control	Intervention	Difference^#^	*p*	Control	Intervention	Difference^#^	*p*
Knowledge (mean ± SD)								
Signs and symptoms of malaria	3.58 ± 1.73	3.86 ± 1.64	0.29 (− 0.12, 0.69)	0.163	3.46 ± 1.30	6.81 ± 1.43	3.35 (3.03, 3.68)	< 0.001*
Malaria diagnosis and treatment	2.52 ± 1.11	2.74 ± 1.44	0.22 (− 0.91, 0.53)	0.165	3.07 ± 1.44	6.49 ± 1.28	3.42 (3.09, 3.74)	< 0.001*
Attitude (mean ± SD)								
Severity of malaria	5.15 ± 1.40	5.20 ± 1.09	0.05 (− 0.25, 0.35)	0.724	4.93 ± 1.29	7.84 ± 1.83	2.91 (2.52, 3.29)	< 0.001*
Transmission of malaria	3.86 ± 1.84	3.99 ± 1.84	0.13 (− 0.31, 0.57)	0.559	4.06 ± 1.50	7.74 ± 1.29	3.67 (3.34, 4.01)	< 0.001*
Practice (mean ± SD)								
Proper use of LLINs	4.02 ± 1.10	4.11 ± 1.40	0.09 (− 0.21, 0.40)	0.551	3.82 ± 1.49	7.78 ± 0.96	3.96 (3.67, 4.26)	< 0.001*
Care-seeking	3.89 ± 1.37	3.75 ± 1.47	− 0.14 (− 0.48, 0.20)	0.412	3.92 ± 1.30	7.50 ± 1.21	3.58 (3.28, 3.88)	< 0.001*
Treatment adherence	3.48 ± 1.62	3.34 ± 1.42	− 0.15 (− 0.51, 0.22)	0.421	3.18 ± 1.68	7.79 ± 1.48	4.61 (4.23, 4.99)	< 0.001*

*LLINs* long-lasting insecticide treated nets

* Significance at unpaired t-test with *p*-value < 0.05; ^#^mean (95% CI)

The overall attitude and practice scores showed similar trends; there was no difference between the two groups during the pre-intervention assessment, but a statistically significant difference was found in the post-intervention assessment (*p *< 0.001). The mean scores ± SD in the intervention group were 7.84 ± 1.83 for attitude toward severity of malaria and 7.74 ± 1.29 for transmission of malaria, whereas the scores for these variables remained equivalent to the baseline scores in the control group (4.93 ± 1.29 and 4.06 ± 1.50, respectively).

The post-intervention mean scores in all three categories of good practice, specifically utilization of LLINs, malaria care-seeking, and treatment adherence, improved from 4.11 ± 1.40, 3.75 ± 1.47 and 3.34 ± 1.42 to 7.78 ± 0.96, 7.50 ± 1.21, and 7.79 ± 1.48, respectively. In the control group, the mean scores for these variables remained equivalent to the baseline mean scores (3.82 ± 1.49, 3.92 ± 1.30 and 3.18 ± 1.68, respectively).

### Within-group comparison

In addition to group comparisons, the within-group results (participants involved in both the control and intervention groups for the pre-and post-intervention assessment) were also differentiated (Table 3). The post-intervention assessment revealed statistically significant differences (*p *< 0.001) in all variables for the intervention group. Unexpectedly, the post-intervention knowledge scores for the diagnosis and treatment of malaria were also significantly higher in the control group in comparison to the baseline scores (*p *< 0.05). Yet, the improvement in the scores was much greater in the intervention group than in the control group (2.52 ± 1.11 to 3.07 ± 1.44 in the control group and 2.74 ± 1.44 to 6.49 ± 1.28 in the intervention group).

**Table 3:** Mean scores of knowledge, attitude and practice within the control and intervention groups before and after intervention

Descriptions	Control	*p*	Intervention	*p*
	Difference^#^		Difference^#^	
Knowledge				
Signs and Symptoms of malaria	0.12 (− 0.26, 0.49)	0.544	− 2.95 (− 3.31, − 2.59)	< 0.001*
Malaria diagnosis and treatment	− 0.55 (− 0.86, − 0.23)	0.001*	− 3.74 (− 4.06, − 3.42)	< 0.001*
Attitude				
Severity of malaria	0.22 (− 0.11, 0.54)	0.198	− 2.63 (− 2.99, − 2.28)	< 0.001*
Transmission of malaria	− 0.2 (− 0.61, 0.21)	0.338	− 3.74 (− 4.12, − 3.37)	< 0.001*
Practice				
Proper use of LLINs	0.2 (− 0.12, 0.52)	0.220	− 3.67 (− 3.95, − 3.39)	< 0.001*
Care-seeking	− 0.3 (− 0.36, 0.30)	0.853	− 3.75 (− 4.07, − 3.43)	< 0.001*
Treatment adherence	0.31 (− 0.10, 0.71)	0.134	− 4.45 (− 4.79, − 4.11)	< 0.001*

* Significance at unpaired t-test with *p*-value < 0.05; ^#^mean (95% CI)

### Confirmed malaria cases

Correlated with the intervention, a large declining trend for malaria morbidity was also noticed in the intervention group. The confirmed cases per tested population in the same period of two adjacent years illustrated a reduction in the malaria burden (Table 4). The outcome affirmed that the proportion of malaria cases dropped significantly to 2% after the six-month intervention in comparison to 12% in the control group (*p *< 0.05). Again, a significant decrease in morbidity was found (from 17 to 2%) when correlated with the intervention group itself; however, smaller change in morbidity was found for the control group (18% to 12%) after 6 months.

**Table 4:** Comparison of malaria morbidity (percentage of confirmed cases) during same period in 2017 and 2018

June–November, 2017 (%)				June–November, 2018 (%)				Between groups (2017 vs 2018)			
								Control		Intervention	
Intervention	Control	χ^2^	*p*	Intervention	Control	χ^2^	*p*	χ^2^	*p*	χ^2^	*p*
17	18	0.103	0.860	2	12	11.527	0.001*	1.970	0.205	18.455	< 0.001*

Data source: unpublished data from respective township’s vector borne disease control team

*p*-value by Fisher’s exact test, * significance at *p*-value < 0.05

### Attitude towards the intervention

In addition to measuring intervention effect, it is also important to assess the community’s attitude regarding this type of intervention when considering future implementation. The majority of participants (> 80%) had positive opinions regarding the loudspeaker announcements as an intervention, and preferred continuing the announcements (Table 5). In contrast, a small proportion (8.6%) reported having to endure increased noise and unclear announcements.

**Table 5:** Participants’ attitudes toward the loudspeaker announcement intervention (n = 140)

Descriptions	Frequency	Percentage
Like to be continued	116	82.9
Suffered noise pollution or unclear messages from loud-speakers^a^	12	8.6
Well absorbed and clearly heard information	109	77.9
The provided messages reminded me to have blood test soon after having fever	111	79.3
The intervention was good for me and other villagers	122	87.1

^a^Negative statement

## Discussion

This study was implemented among people living in a malaria-endemic area with allocated VMWs during the peak malaria transmission season. The baseline KAP findings revealed very low-level scores in the four control and intervention villages. With basic community-based malaria control activities already in place, one might suspect that villagers’ mindsets are difficult to change, resulting in consistently poor levels of KAP [28]. The low KAP scores, despite the presence of VHWs in these communities suggest that VHWs may not be functioning effectively, possibly because of inadequate training or support, or other multi-dimensional factors, such as incentive schemes, or lack of community and family support [29].

Although more than 90% of households owned sufficient LLINs (2 persons/net) in both groups, participants reported very low levels of proper LLIN utilization at the baseline, similar to a national-level study in Myanmar that showed poor LLIN utilization among caregivers as well as children [30]. Previous studies suggest the quality of the mosquito nets, especially the types of lace used to ensure the privacy of women and children, as well as the color and durability of the nets might affect its utilization [5]. Traditional beliefs as well as low educational levels may also influence LLIN usage [31]. One other issue that a Kenyan study reported was that people do not sleep under nets during the total duration of the malaria vectors’ ordinary biting cycle (i.e. dusk to dawn) [32]. In this study, a profound increase was observed in the utilization rate in the intervention group with a difference in the mean scores ranging from 3.67 to 4.26.

An additional finding of this baseline study was that most subjects exhibited poor malaria care-seeking behaviour. All the malaria-related services are free of charge, irrespective of the patients’ abilities to pay for a consultation. As the malaria-related mortality and overall malaria burden declined throughout the decades, the community’s attitude towards the severity of the disease and its causation has decreased accordingly [33]. Thus, the refreshing knowledge about severe malaria delivered by the loudspeaker announcements may help alert the community for potential occurrence of severe malaria if it is neglected.

The intervention reported here using loudspeaker announcements of health messages about malaria prevention and treatment resulted in improved KAP scores among household participants. As a result of repeated announcements, a larger span of the community had increased awareness of malaria. This finding is in line with several other studies, in which increased community KAP levels were verified by the use of an intervention [27, 34, 35]. As part of the increasing KAP levels, unexpectedly, knowledge levels about the diagnosis and treatment of malaria among participants from the control group also were boosted after a six-month period. This outcome might be due to the dissemination of information through various media sources (i.e. television, radio, and health education videos) potentially from neighbouring villages [36]. In Myanmar, a project has been broadcasting malaria diagnosis and treatment information since 2017 [37]. In addition, though buffer zones existing between control and treatment village should prevent from message contamination, it cannot be completely excluded due to reasons such as relative-visiting activities between control and intervention villages.

To further assess the efficacy of the loudspeaker announcement intervention, malaria incidences throughout the intervention months were compared to the same period from the previous year. A significant reduction in morbidity (from 17 to 2%) was found among members of the community in which the intervention occurred. To halt onward transmission of malaria infection, it is essential to receive early and effective treatment; and 100% adherence to treatment is required to ensure a full cure [4]. The loudspeaker-delivered messages reminded participants to seek timely diagnosis and receive prompt treatment at the first onset of fever. This step might have interrupted indigenous transmission and resulted in a declining trend of malaria among members within the intervention group. In the control villages, a similar trend of reduced malaria morbidity (from 18 to 12%) was observed, although the degree of decrease was much less than that in the intervention group. A decreasing trend is consistent with the overall decline of malaria incidence in the entire country, as Myanmar is progressing towards malaria elimination. Previous studies have reported similar findings wherein increasing community awareness resulted in a decrease in disease burden [27, 28, 38]. Moreover, improvements in proper LLINs utilization can have an impact on malaria transmission [39, 40].

In terms of participants’ satisfactions with the type of intervention, most participants were satisfied with the loudspeaker intervention. However, a few complained about noise pollution, and participants residing on the periphery of the villages were not consistently able to decipher the broadcasted messages. Interference from the surrounding environment may be a potential reason. One study noted that intervention implementers should be aware of environmental and human factors that might disrupt the intervention [41]. Meanwhile, residents surrounding the intervention source (i.e. houses with loudspeakers) may have been impacted by increased noise the most. Nevertheless, future implementation should focus on reducing identified complaints (for example, using more loudspeakers to cover the peripherals of the village, but reducing the volume of individual speakers).

Overall, this intervention was effective in delivering messages, was well accepted by the community, helped improve KAP levels and was linked to reduction of malaria morbidity. However, when implementing a new intervention, it is important to assess its sustainability by considering its cost, complexity of implementation, and maintenance. In this case, the total cost for a loudspeaker system was approximately 200 USD. Most populations in rural areas are used to such a loudspeaker system, which they can maintain by themselves with low fees. Future studies should target areas with larger populations and evaluate the effect of the intervention after a three-month, six-month and one-year interval.

This study has a few limitations. Additional factors may influence the quality and interpretation of the data. For example, villagers may obtain malaria-related information from other sources such as radio and television, which may result in overall improvement of KAP scores in all villages. Although this may be possible, it will unlikely have a major impact on the outcome since control and intervention villages should be affected similarly. This study was conducted in a six-month period, and the result may differ with long-term intervention. Because of the quasi-experimental study design, the assignment of the control and intervention villages may not be fully random. Thus, the results of this study might not be generalized for all the locations in Myanmar. Other follow-up assessments to explore the long-term effectiveness in different sites are warranted. Other factors may also be involved to contribute to the overall reduction of malaria morbidity in both control and treatment villages.

## Conclusions

The delivery of loudspeaker-based health announcements in the high malaria transmission season resulted in considerable improvement of overall KAP scores in a malaria-endemic region in northern Myanmar. These improved scores were associated with a greater decline in malaria cases among the intervention villages, suggesting that this intervention might be effective among these rural locales for reducing the malaria burden. The national malaria control programme may consider implementing this user-friendly, low-cost intervention and provide supports for its sustainability. Participation from the local community should be encouraged to broaden the coverage area. Messages not only for malaria but also other preventable diseases may be included.

## Data Availability

The datasets used and/or analyzed during the current study are available from the corresponding authors on reasonable request.

## Abbreviations

WHO: World Health Organization
GMS: Greater Mekong Sub-region
LLIN: long-lasting insecticide-treated nets
VMW: village malaria worker
VBDC: vector borne disease control
MMK: Myanmar Kyats
MoHS: Ministry of Health and Sports
SD: standard deviations
API: Annual Parasite Incidence
KAP: knowledge, attitude, and practice
